# Imaging of brain clearance pathways via MRI assessment of the glymphatic system

**DOI:** 10.18632/aging.205322

**Published:** 2023-12-26

**Authors:** Yi He, Jitian Guan, Lingfeng Lai, Xiaolei Zhang, Beibei Chen, Xueqing Wang, Renhua Wu

**Affiliations:** 1 Department of Medical Imaging, Second Affiliated Hospital, Shantou University Medical College, Shantou, China; 2 Department of Ultrasound, Shantou Central Hospital, Shantou, Guangdong, China

**Keywords:** aging, chemical exchange saturation transfer, deep cervical lymph node ligation, glymphatic system, magnetic resonance imaging

## Abstract

Glymphatic clearance dysfunction may play an important role in a variety of neurodegenerative diseases and the progression of ageing. However, *in vivo* imaging of the glymphatic system is challenging. In this study, we describe an MRI method based on chemical exchange saturation transfer (CEST) of the Angiopep-2 probe to visualize the clearance function of the glymphatic system. We injected rats with Angiopep-2 via the tail vein and performed *in vivo* MRI at 7 T to track differences in Angiopep-2 signal changes; we then applied the same principles in a bilateral deep cervical lymph node ligation rat model and in ageing rats. We demonstrated the feasibility of Angiopep-2 CEST for visualizing the clearance function of the glymphatic system. Finally, a pathological assessment was performed. Within the model group, the deep cervical lymph node ligation group and the ageing group showed higher CEST signal than the control group. We conclude that this new MRI method can visualize clearance in the glymphatic system.

## INTRODUCTION

The paravascular pathway, also known as the “glymphatic” pathway, is a recently identified waste removal system in the brain. It is associated with the insoluble amyloid plaques, ageing and the development of neurodegenerative diseases [1–7]. According to the glymphatic system theory, toxic substances are cleared by interstitial fluid, which flows out through perivenous channels, carries the brain’s metabolic “waste” to the cerebrospinal fluid pool, and drains from the meningeal lymphatics to the cervical lymphatics. Impairment of this system is thought to affect the clearance of brain metabolites (amyloid β, tau proteins, etc.) [4, 8, 9]. The glymphatic system is influenced by vascular pulsation [10], sleep [11, 12], age [4, 13] and other factors, and a decline in its function may be associated with ageing, AD and other neurodegenerative pathologies [5, 14–16].

However, in clinical practice, imaging the glymphatic system has always been challenging, which greatly restricts research on and diagnosis of the glymphatic system. Fluorescence imaging is commonly used in early laboratories to study the glymphatic system. Recently, contrast-enhanced MRI with gadolinium-based contrast agents (GBCAs) has been increasingly used in live animals and clinical studies because of its high resolution, high penetration, and relatively low invasiveness [17, 18]. However, GBCAs have a limited ability to cross the blood-brain barrier, and the clinical assessment of the glymphatic clearance function requires the contrast agent to be injected intrathecally, which is a complicated operation; furthermore, GBCA remains in the body for long periods, creating a risk of residual gadolinium deposition in the brain [19, 20]. The blood-brain barrier protects the brain but also creates difficulties in diagnosis and treatment. Many scholars have attempted to develop new MRI techniques to noninvasively assess the function of the glymphatic system. Recent studies have also found that some nanomedicines are cleared through paravascular pathways [21, 22]. Angiopep-2 is a peptide-based probe; compared with large molecules such as proteins, peptides in general have better *in vivo* stability and lower immunogenicity and are less expensive [23, 24]. Additionally, Angiopep-2 penetrates the blood-brain barrier by binding to low-density lipoprotein receptor-related protein 1 (LRP-1) [25]. This probe can be used for intracerebral imaging, and fluorescence imaging confirms that it can penetrate the blood-brain barrier [26]. This artificial peptide penetrates the blood-brain barrier through low-density lipoprotein receptor-related protein 1 (LRP-1) and has been used in clinical trials [25, 27].

Chemical exchange saturation transfer (CEST) imaging is a novel molecular imaging technique that allows the noninvasive tracking of substances at the molecular level. Compared with magnetic resonance spectroscopy (MRS), CEST has better spatial resolution and detects compounds at lower concentrations [27–29]. Previous studies in our laboratory found that Angiopep-2 could serve as a novel exogenous probe for CEST MRI [30], with the strength of the signal depending on the number of protons exchanged per molecule and the presence of -NH or -OH proton exchange sites. The CEST effect of Angiopep-2 appears at 3.2 ppm. To deal with the difficulty in imaging the glymphatic system due to the presence of the blood-brain barrier, we attempted to evaluate the glymphatic system by intravenous injection of Angiopep-2 and observe the metabolism of exogenous probes in the brain through CEST-MRI.

Here, we used Angiopep-2 as a probe to assess glymphatic clearance function in two rat models of impaired glymphatic function, namely, bilateral deep cervical lymph node ligation (DCLN) and ageing (OLD), compared to rats with normal glymphatic function. For this purpose, Angiopep-2 was injected into the rats via the tail vein, and the changes in its concentration in the brain were observed by CEST MR.

## RESULTS

### *In vivo* imaging

**Figure 1:**
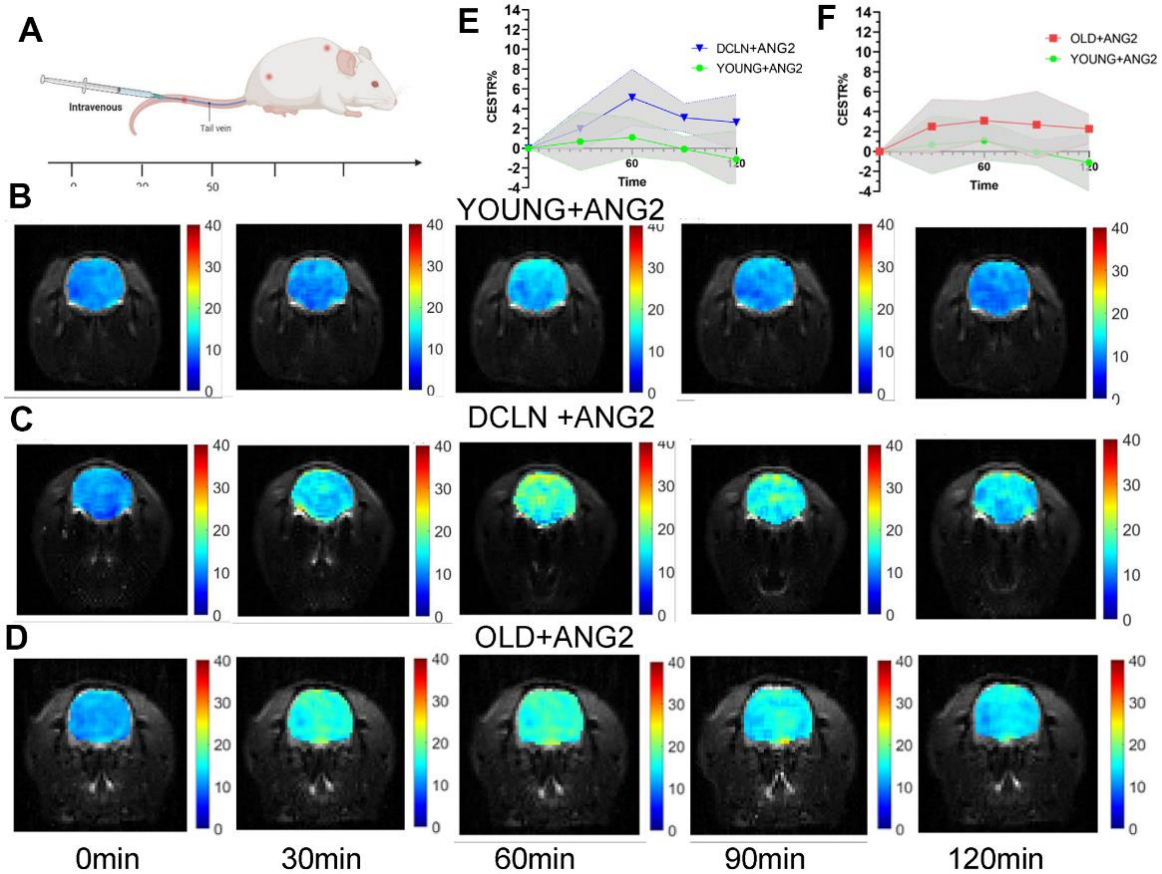
**Angiopep-2 CEST MRI results for YOUNG and DCLN rats.** (**A**) Intravenous injection of Angiopep-2 and measurement of CEST values at 0, 30, 60, 90, and 120 min. (**B**–**D**) Representative images for YOUNG, DCLN and OLD rats injected with Angiopep-2 (ANG2). (**E**) Comparison between YOUNG and DCLN rats (n=5). (**F**) Comparison between YOUNG and OLD rats (n=5).

CEST-MRI was performed at different time points after injection of Angiopep-2 in untreated YOUNG Sprague–Dawley (SD) rats (aged 2-3 months) and in DCLN SD rats (Figure 1A). Unified selection of hippocampal level scanning. Figure 1B–1D shows representative images from YOUNG, DCLN and OLD (14-17 months of age) rats after injection of Angiopep-2. These images were acquired 0, 30, 60, 90, and 120 min after injection. Figure 1E shows the brain Angiopep-2 CEST ratio (CESTR) curves (DCLN and YOUNG) after injection of Angiopep-2, starting at baseline (0 min). Figure 1F shows the brain Angiopep-2 CESTR curves (OLD and YOUNG) after Angiopep-2 injection. These results showed that DCLN and OLD rats had greater enhancement after injection of Angiopep-2 than normal rats. The CESTR was gradually enhanced at 30-60 min, with the strongest effect at 60 min, and gradually diminished with time.

**Figure 2:**
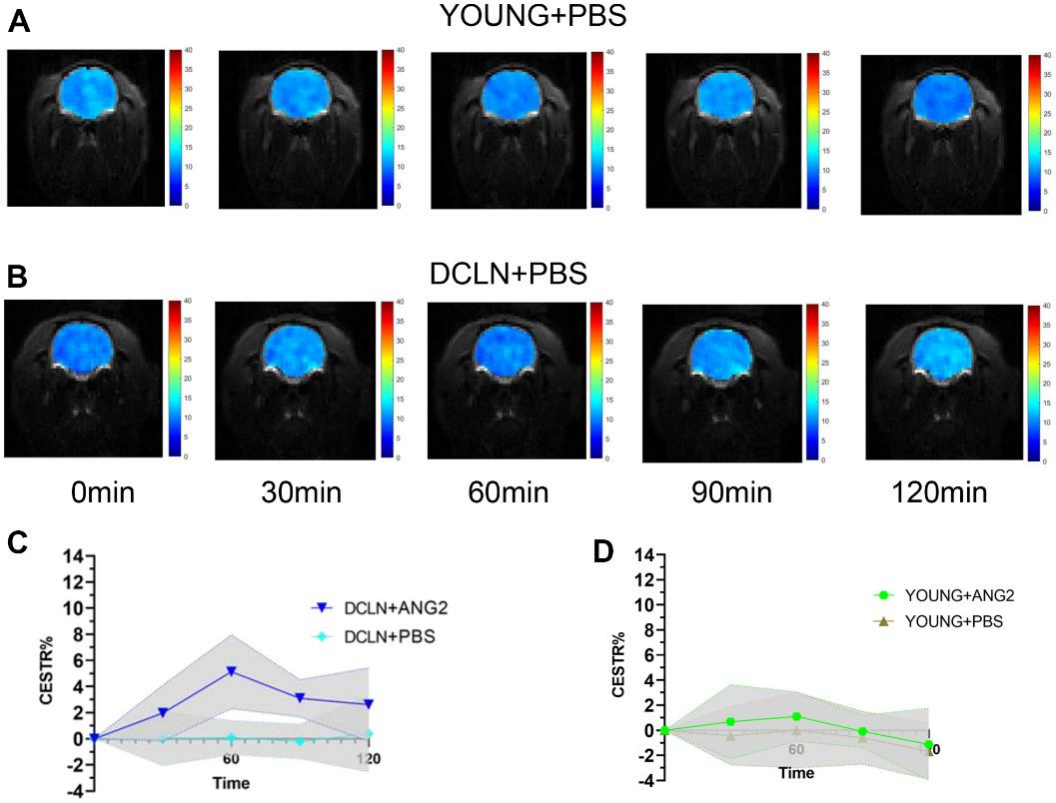
**Angiopep-2 CEST MRI results.** (**A**, **B**) Representative images for YOUNG and DCLN rats injected with PBS. (**C**) Comparison of YOUNG rats injected with ANG2 and PBS (n=5). (**D**) Comparison of DCLN rats injected with ANG2 and PBS (n=5).

**Figure 3:**
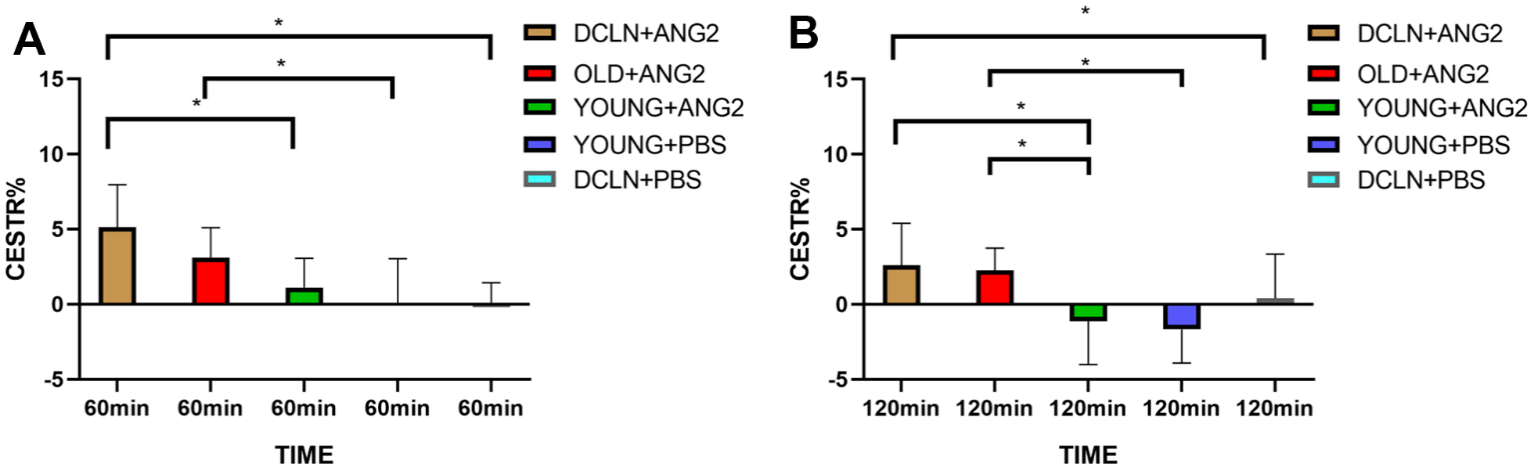
(**A**) CESTR values in the five groups at 60 min after injection (n=5). (**B**) CESTR values in the five groups at 120 min after injection (n=5). Significance level: *P < 0.05.

Figure 2A, 2B shows representative images of YOUNG and DCLN rats after phosphate-buffered saline (PBS) injection. These images were acquired 0, 30, 60, 90, and 120 min after injection. Figure 2C, 2D shows the brain Angiopep-2 CESTR curves (DCLN and YOUNG) after PBS injection, starting at baseline (0 min). Figure 3A, 3B shows that the CESTR peaked at 60 min in all three groups of Angiopep-2-injected rats. The CESTR increased less over the course of 60 min in YOUNG+ANG2 rats than in rats with glymphatic damage (DCLN+ANG2), P<0.05. Among PBS-injected rats (YOUNG+PBS, DCLN+PBS), there was a significant difference between DCLN+ANG2 and OLD+ANG2 rats, P<0.05, with DCLN rats having a higher CESTR. Compared to that of YOUNG+PBS rats, the signal of YOUNG+ANG2 rats was increased, but there was no significant difference. At 120 minutes, the CESTR values in DCLN+ANG2 and OLD+ANG2 rats were higher than in YOUNG and PBS rats, P<0.05.

### Fluorescence staining of brain tissue

**Figure 4:**
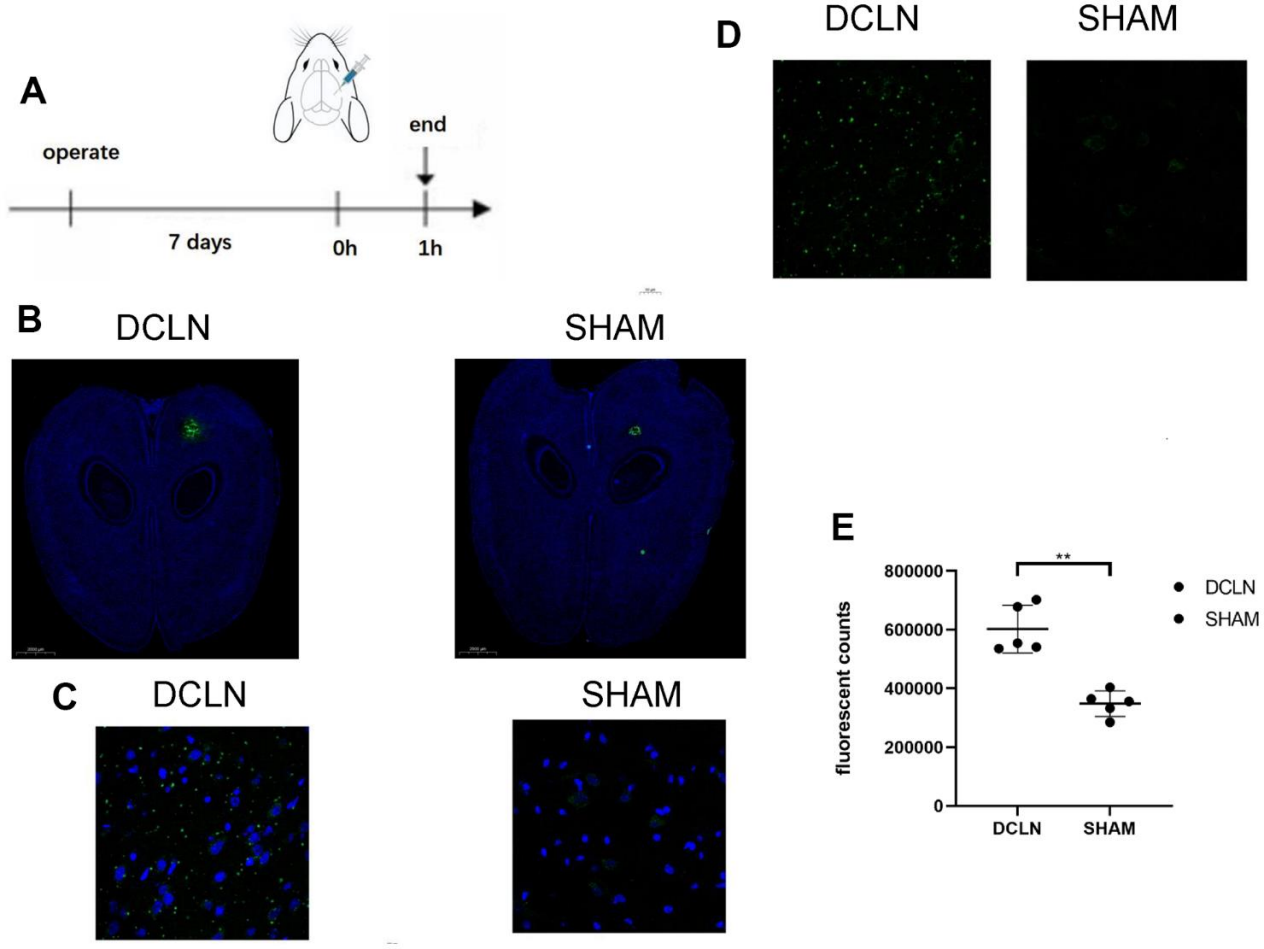
**Effect of an impaired glymphatic system on Angiopep-2 efflux from the brain.** (**A**) Seven days after bilateral DCLN ligation, 5 μl of FITC-Angiopep-2 (0.05 mg/μl, AP -5.0, ML 2.0, DV 3.0) was stereotaxically injected (AP, -5.0 mm; ML, −2.0 mm; DV, +3.0 mm) into the brain parenchyma. (**B**) Representative brain sections of the two groups (DCLN and SHAM; green, FITC-Angiopep-2; blue, DAPI, 2000 μm). (**C**, **D**) Confocal microscope imaging of representative brain sections (DCLN and SHAM; green, FITC-Angiopep-2; blue, DAPI, 50 μm). (**E**) Quantification of postinjection FITC-Angiopep-2 fluorescent counts in the brain tissue of the two groups. Significance level: **P < 0.05.

Figure 4A shows FITC-Angiopep-2 injection in the brain parenchyma of DCLN and SHAM SD rats; the rats were sacrificed 1 h after injection, and the brain slides were examined. Figure 4B shows representative fluorescence images of whole-brain sections (green, FITC-Angiopep-2; blue, DAPI; n=5). More FITC-Angiopep-2 remained in the DCLN group than in the SHAM group, indicating that the former group had an impaired glymphatic system and a reduced Angiopep-2 clearance rate; Figure 4E illustrates this difference in residual Angiopep-2 was significant, P<0.05. Figure 4C, 4D shows representative confocal images (green, FITC-Angiopep-2; blue, DAPI). The DCLN group had more FITC-Angiopep-2 residue than the SHAM group, especially in the intercellular space.

### Pathology

**Figure 5:**
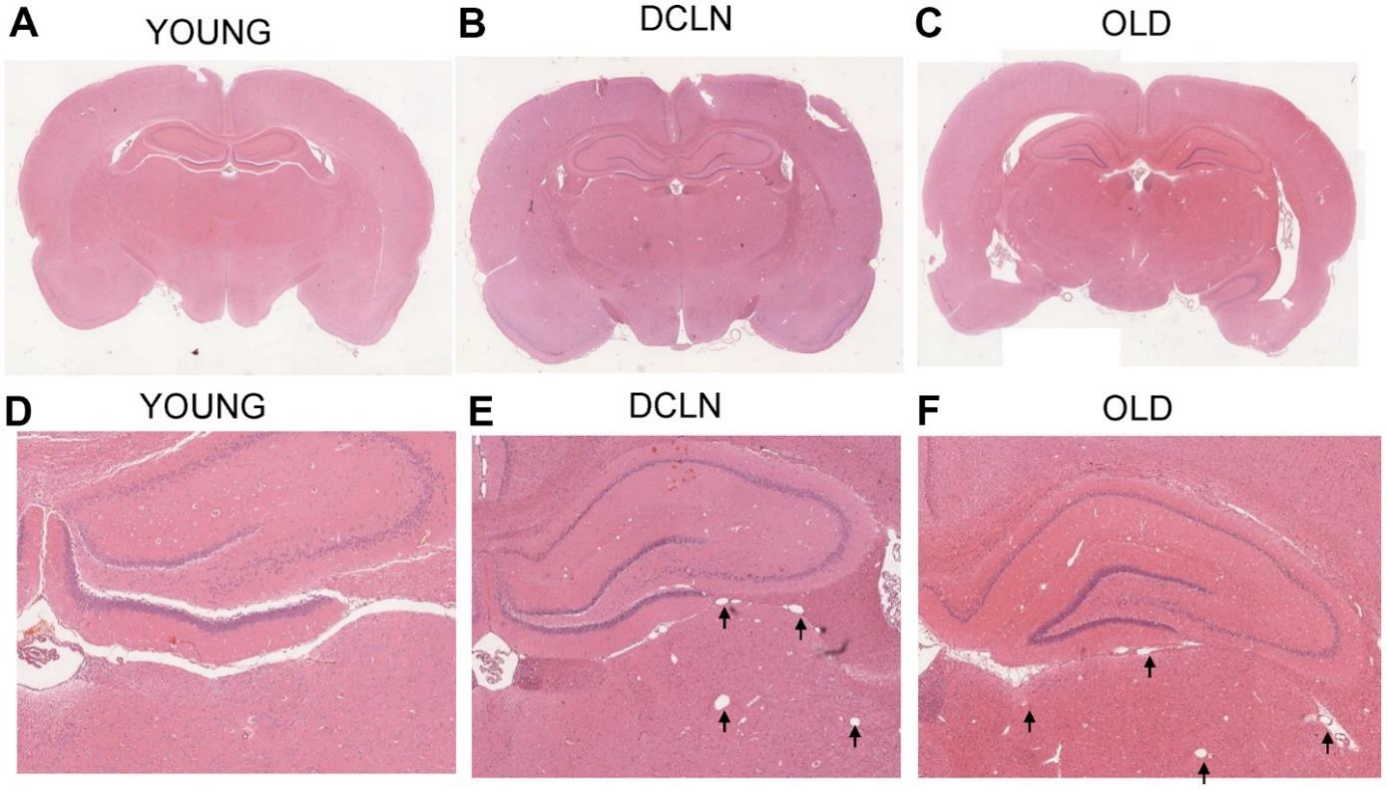
HE-staining histology results from YOUNG (**A**, **D**), DCLN (**B**, **E**), and OLD (**C**, **F**) SD rats. The black arrows indicate the dilated perivascular spaces in the brain.

Figure 5A–5C shows haematoxylin and eosin (HE)-stained sections of the brains of YOUNG, OLD, and DCLN SD rats after Angiopep-2 injection, respectively; no significant tissue destruction was seen anywhere in the brain, suggesting that Angiopep-2 is relatively safe. Figure 5D, 5E shows local images from the three groups of rats; more dilated blood vessels and perivascular gaps can be seen in the DCLN and OLD rats than in the YOUNG group.

### Behavioural tests

**Figure 6:**
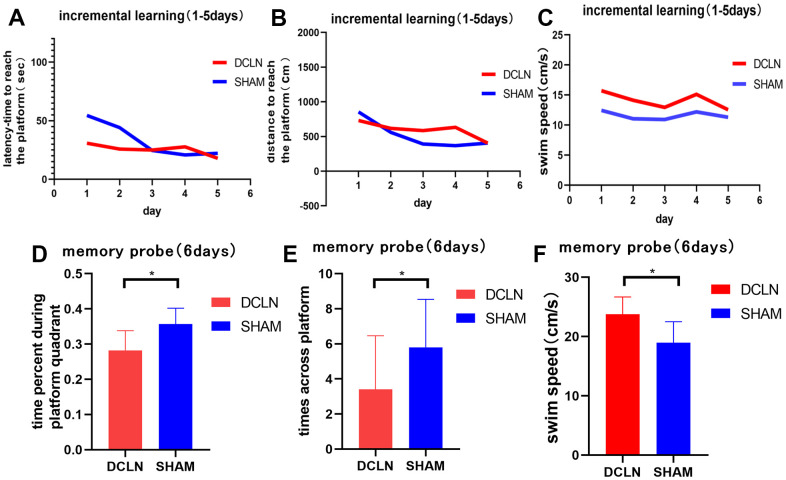
**Morris water maze.** (**A**, **B**) The spatial learning and memory test began on postoperative day 8; the graphs indicate the latency and distance for both groups during the 5-d training period. (**C**) Swimming speed differed between the DCLN model group and SHAM group (P<0.05). (**D**–**F**) Time in the correct quadrant, number of transits across the former platform location, and swimming speed in the two groups in the memory probe test on postoperative day 13. Significant differences were found between the DCLN model group and the SHAM group. Significance level: *P<0.05.

Spatial learning was tested with the Morris water maze over a 5-d period starting 8 d after surgery, followed by a memory trial on day 6. Significant differences in swimming speed were found between the model and sham groups (P<0.05, Figure 6C, 6F), implying a systematic bias in latency (Figure 6A). The distance swum by each group gradually decreased over the course of training. The distance travelled to reach the platform was greater in the DCLN model group than in the SHAM group, but there was no significant difference between them (P>0.05, Figure 6B). The results suggests that the DCLN model group may have had a decline in spatial memory compared to the SHAM group as time passed. Overall, during the memory test, DCLN model rats spent significantly less time in the correct quadrant and crossed the platform less frequently than SHAM rats (P<0.05, Figure 6D, 6E).

## DISCUSSION

In this study, we found elevated CESTR values in rats with impaired glymphatic system clearance function by comparing the dynamic Angiopep-2 CESTR profile in the brains of DCLN, OLD and normal SD rats after intravenous injection of Angiopep-2. The results suggested that damage to the glymphatic clearance system impedes the clearance of Angiopep-2, resulting in more Angiopep-2 remaining in the brain. We demonstrated the feasibility of using CEST MRI for the functional assessment of glymphatic clearance. To demonstrate that impaired clearance of the glymphatic system leads to a slowing of Angiopep-2 clearance, we conducted an experiment in which FITC-Angiopep-2 was injected into the brain parenchyma of impaired and normal glymphatic-model rats. Our findings revealed slowed Angiopep-2 clearance following impairment of clearance in the glymphatic system.

In this study, OLD and DCLN rats showed higher CESTR values than YOUNG rats after Angiopep-2 injection. First, damage to the clearance function of the glymphatic system leads to greater Angiopep-2 outflow obstruction in the brain, thus resulting in an increased Angiopep-2 signal. It has been shown that damage to the glymphatic system causes a decrease in clearance [31, 32]. Second, our findings may be related to the destruction of the blood-brain barrier, which has been shown to be damaged in neurodegenerative disorders and ageing, and breakdown of the blood-brain barrier is related to cognitive decline [33–35]. The effect of the CESTR in DCLN rats was higher than that in OLD rats, possibly because ligation of the lymph nodes leads to brain oedema, which further affects the flow of the glymphatic system [36]. Brain oedema may further impact the flow of fluid in the brain, causing further slowing of Angiopep-2 clearance [37, 38]. The flow of fluid in the brain is related to the clearance function of the glymphatic system [10]. Additionally, a study found that improving the function of meningeal lymphatic vessels promoted brain oedema [39]. These results fully indicate that neurodegenerative disorders and ageing not only affect and lead to more drugs entering the blood-brain barrier but also have defects in related clearance functions. The above studies indicated that consequently, more drugs (such as Angiopep-2) enter the brain, while more drugs are blocked from flowing out of the brain. In our experiment, the increased level of Angiopep-2 caused an increase in signal.

We injected the fluorescent reagent FITC-Angiopep-2 into the brain of DCLN and SHAM model rats to simulate the metabolism of Angiopep-2 in the brain parenchyma. By comparing the residual amount of FITC-Angiopep-2 in the brain parenchyma of DCLN rats with that in rats with glymphatic system damage and SHAM rats, the clearance of Angiopep-2 after glymphatic system damage was demonstrated. Under confocal microscopy, it was found that after 1 hour of intracerebral metabolic transport, the fluorescently labelled Angiopep-2 remaining in the tissue of rats in the SHAM group was mainly distributed in the cytoplasm near the nucleus, and very little remained in the intercellular fluid. Fluorescence protein remained in both the interstitial fluid and cytoplasm of DCLN rats, and more fluorescently labelled Angiopep-2 remained in the interstitial fluid of DCLN than that of SHAM. Iliff et al. found that the glymphatic system in the brain mainly secretes interstitial fluid into the perivascular space through astrocyte aquaporin-4, after which it is gradually cleared out of the brain [32]. In the brain tissue of DCLN rats, more Angiopep-2 remained in both the interstitial fluid and cytoplasm, suggesting that more Angiopep-2 was prevented from entering the interstitial fluid due to impaired transport between the interstitial fluid and the perivascular space caused by damage to the glymphatic system. At the same time, the inflow of cerebrospinal fluid caused by cervical lymph ligation also affects the outflow of Angiopep-2 from the interstitial fluid [36].

Imaging of the glymphatic system has been studied extensively. In 2013, Iliff et al. first applied dynamic contrast-enhanced MRI in imaging the glymphatic system [17] because it allows accurate, sensitive, long-term observation of multiple levels of brain tissue and deep structures with relatively low risk, Resulting in its gradual use in clinical practice [18]. It is the most widely accepted technique and has been developed and applied in recent years for the imaging of meningeal lymphatic vessels [40]. However, it is difficult for GBCAs to penetrate the blood-brain barrier; instead, they require lumbar puncture injection followed by 24-48 h of observation [41–43]. PET is mainly used in the study of glymphatic system to assess amyloid β and tau proteins [44, 45]. The most commonly used PET probes are generally labelled with 11C and 18F; these radionuclides have a half-life of 20-118 min, and a certain amount of time is needed to observe the clearance rate.

Some scholars have noninvasively assessed the glymphatic system by measuring changes in the perivascular diffusion velocity of water molecules by DWI [46, 47]. For example, Ohene et al. measured the rate of entry of labelled water molecules into the brain by arterial spin labelling (ASL) to assess the function of the glymphatic system [48]. Huang et al. assessed the impaired glymphatic system in Alzheimer disease model rats by observing a slowed rate of glucose clearance in the cerebrospinal fluid pool [49]. Chen et al. previously used lymphatic CEST to assess the function of the glymphatic system [50]. The experiment used Angiopep-2 as an exogenous contrast agent, which can yield high-contrast noise while avoiding the influence of the difference in initial metabolic concentration.

Unlike experimental conditions, patients cannot tolerate two hours of continuous scans. In clinical applications, the number of scans can be reduced, such as by scanning at only three time points, for example 0, 60, and 120 min, to reduce the scanning time. In our experiment, a slightly higher dose was selected to obtain a better Angiopep-2 CEST effect. However, in clinical applications, the total injection volume is limited; therefore, the dissolved concentration of Angiopep-2 should be increased, and the injection should be carried out with less liquid to meet the needs of the clinical examination. The CEST effect of Angiopep-2 occurs at 3.2 ppm, which is closer to the amide proton transfer (APT) effect and easily disturbed. In future studies, the concentration of Angiopep-2 should be modified appropriately in order to obtain greater and more specific effects. In this experiment, due to the limitation of scanning time, only a single layer was scanned by CEST-MRI. Subsequent experiments should optimize the CEST sequence to reduce the scanning time, and more brain information can be obtained by applying 3D-CEST sequence for whole-brain scanning.

## MATERIALS AND METHODS

All reagents are commercially available and could be used without further purification. Angiopep-2 was synthesized by ChinaPeptides Co., Ltd. (Shanghai, China), peptide sequence TFFYGGSRGKRNNFKTEEY, molecular weight 2301.48, chemical formula C104H149N29O31. FITC-Angiopep-2 (FITC-TFFYGGSRGKRNNFKTEEY) was synthesized by GL Biochem Ltd. (Shanghai, China), molecular weight 2804.00.

Rats were provided by the Animal Center, who purchased them from Shantou University School of Medicine, Guangdong, China. The rats were anaesthetized with isoflurane vapour; specifically, 4.0% isoflurane was used for anaesthesia induction, and 1.5-2.5% isoflurane was used for maintenance. Body temperature and respiration were continuously monitored using an MRI-compatible small-animal monitoring system (SAI Technologies, Memphis, TN, USA).

### MRI experiments

All experiments were performed on a 7.0 T horizontal bore small-animal MRI scanner (Agilent Technologies, Santa Clara, CA, USA) with a horizontal bore size of 16 cm and standard 9563 coils (Agilent Technologies, Santa Clara, CA, USA, USA) and surface coils (Timemedical Technologies, Shanghai, China) for transmission and reception.

In the CEST imaging experiments, an echo-planar imaging (EPI) readout sequence with continuous wave saturation (CW-EPI) was used to obtain the Angiopep-2 z spectrum and perform CEST imaging. The scanning parameters were as follows: saturation duration = 3 s, B1 = 3 μT, layer thickness = 2 mm, field of view (FOV) = 40 × 30 mm, matrix size = 64 × 64, repetition time (TR) = 6000 ms, echo time (TE) = 25 ms, and bandwidth = 267 000 Hz, with 122 frequency offsets unevenly distributed from −6 to 6 ppm relative to the resonance of water. The total scanning duration was 13 min and 30 s.

### *In vivo* imaging in SD rats

Twenty-five SD rats were scanned (YOUNG, 2-3 months old, n=5; OLD 14-17 months old, n=5; DCLN model group, 2-3 months old, n=5; YOUNG+PBS group, 2-3 months old, n=5; and DCLN+PBS group, 2-3 months old, n=5). Then, Angiopep-2 (30 mM, 1 mL) or PBS (1 mL) was injected into the tail vein of the rats. After injection, the rats were scanned at different time points (0, 30, 60, 90, and 120 min) and divided into five groups: Angiopep-2-injected young SD rats (YOUNG+ANG2, 2-3 months old, n = 5), PBS-injected young rats (YOUNG+PBS, 2-3 months old, n = 5), Angiopep-2-injected SD rats (OLD, 14-17 months of age, n = 5), Angiopep-2-injected, operated SD rats (DCLN+ANG2, 2-3 months of age, n = 5). and PBS-injected, operated SD rats (DCLN+PBS, 2-3 months of age, n = 5).

### Deep cervical lymph node ligation model

SD rats, weighing 250-300 g, aged 2-3 months were randomly divided into two groups: the DCLN model group (n=5) and the SHAM-operated control group (n=5). Both groups were housed in a location with an ordinary ambient magnetic field. The ambient humidity was controlled at 50 ± 10%, and the temperature was controlled at 22 ± 1 C. The materials needed for surgery included sterilized surgical instruments, alcohol, cotton balls (for preoperative preparation), and No. 8 sutures. Animals were anaesthetized with isoflurane at a concentration of 4%, which was reduced to 2.5-3% after successful induction. The animals were positioned on the operating table with the abdomen facing upwards. After shaving the surgical site and cleaning it with distilled water and iodophor disinfectant, an experimenter made a 5-cm longitudinal incision from the right mandible to the skin of the sternum. The muscles and fascia along the trachea were carefully separated using a blunt-tipped probe. The bilateral deep cervical lymph nodes were then located under the muscles of the sternum, and the associated lymphatic vessels were ligated using a No. 8 suture. SHAM rats underwent the same operation except for the ligation of lymphatic vessels. Finally, the skin, muscles, and fascia were sutured layer by layer and sterilized [51].

### *Ex vivo* analysis

One week after surgery, DCLN rats (n = 5) and SHAM-operated rats (n = 5) were injected with FITC-Angiopep-2 (5 μl, 0.05 mg/μl, AP -5.0, ML -2.0, DV 3.0) in the brain parenchyma. One hour after injection, the animals were euthanized. The brains were removed and stored on ice (-70 C). Brain sections (4 μm) were cut on a cryostat. DAPI was added dropwise to the tissue sections; after 3-5 min of staining, they were rinsed with PBS, sealed with neutral resin, and observed using a Panoramic MIDI microscope and an Ultraview Vox laser confocal microscope.

### HE staining

When all tests were complete, the rats were anaesthetized and perfused with 50 ml of saline and 50 ml of 4% paraformaldehyde via the left ventricle of the heart. At the end of perfusion, the brain was removed, stored in 4% paraformaldehyde for 24 h, dehydrated, embedded in paraffin and histologically examined. Axial sections measuring 5 μm were stained using HE at 50 C for 45 min. All sections were examined neuropathologically under a microscope.

### Morris water maze (MWM)

Seven days after model establishment, all rats were tested in the MWM, which is used to assess spatial learning and memory in rodents. The apparatus consisted of a black, circular pool measuring 150 cm in diameter and divided into four quadrants. The pool contained water at a temperature of 25 ± 1 C, and there was a 10 cm × 10 cm platform hidden below the surface in one of the quadrants, 25 cm from the sidewall. After training, we recorded the time taken by each rat to find the platform (latency), the path it took, and other parameters characterizing the rat’s swim to the platform. The MWM paradigm consisted of four trials per day for 5 consecutive days followed by a probe test on the sixth day. Animals were tested using four trials per day over five consecutive days. The start position changed after each attempt. The animal was gently placed in the apparatus, facing the wall, and allowed to swim for up to 120 s to find the platform. If the animal did not find the platform within 120 s, it was placed there manually and allowed to rest for 10 s. To test memory retention, a probe test involving the removal of the platform was performed 24 h after completion of the platform tests. During the probe test, the preference of the experimental animals for the normal location of the platform was measured. The path that each animal took to the platform in the water was recorded by a video-capture system and transmitted to a computer. The analysis software virtually divided the water maze into its four constituent quadrants and automatically analysed the parameters of path, time, and path time of the animal with respect to these quadrants. Mean swimming speed (cm/s), distance travelled (cm), latency to reach the platform (maximum 120 s), number of transits across the previous location of the platform, and time spent in the correct quadrant (%) were recorded.

### Image and data analysis

All CEST image processing and data analysis were performed in MATLAB (MathWorks, version 8.0, R2021a). On the images, regions of interest were delineated manually. The Lorentz curve was first used to decompose the APT, magnetization transfer (MT), nuclear Overhauser enhancement (NOE), and creatine (Cr) proton pools. The CEST effect was calculated by taking the difference between the proton frequencies of the magnetized intravascular Angiopep-2 and the corresponding reference frequencies at symmetrical positions on opposite sides of the water resonance. The CESTR of Angiopep-2 was calculated using the following equation:

CESTR = (M_−3.2 ppm_ − M_+3.2 ppm_)/M0

where M_±3.2 ppm_ indicates resonance from water at ±3.2 ppm.

### Statistical analyses

All analyses were performed using SPSS 19.0 and GraphPad Prism 8 software. Student’s *t*-test and one-way analysis of variance (ANOVA) were used for the statistical analysis. The significance threshold was set to 0.05.
